# Prediction of generalization of ocular myasthenia gravis under immunosuppressive therapy in Northwest China

**DOI:** 10.1186/s12883-020-01805-1

**Published:** 2020-06-11

**Authors:** Jiaqi Ding, Sijia Zhao, Kaixi Ren, Dan Dang, Hongzeng Li, Fang Wu, Min Zhang, Zhuyi Li, Jun Guo

**Affiliations:** 1 Department of Neurology, Tangdu Hospital, Fourth Military Medical University, Xi’an, 710038 Shaanxi Province China; 2 Intensive Care Unit, Xi’an Fourth Hospital, Xi’an, 710004 Shaanxi Province China; 3 Department of Neurology, Xi’an Children’s Hospital, Xi’an, 710003 Shaanxi Province China

**Keywords:** Myasthenia gravis, Ocular, Generalization, Predictive factor, Immunosuppressive therapy

## Abstract

**Background:**

It is well demonstrated that immunosuppressants can reduce, but not eliminate the risk of generalized development in ocular myasthenia gravis (OMG). In this study, we aimed to explore the predictive factors of generalized conversion of OMG patients who received immunosuppressive treatments.

**Methods:**

OMG patients under immunosuppressive treatments in Tangdu Hospital from June 2008 to June 2012 were retrospectively reviewed. Baseline clinical characteristics were documented. Patients were followed up regularly by face-to-face interview and the main outcome measure was generalized conversion. The logistic regression analysis was performed to determine the predictive factors of generalization of OMG.

**Results:**

Two hundred twenty-three eligible OMG patients completed the final follow-up visit and 38 (17.0%) progressed to generalized MG (GMG) at a median time to generalization of 0.9 year. Patients with adult onset and positive repetitive nerve stimulation (RNS) of facial or axillary nerve had higher conversion rate than those with juvenile onset and negative RNS (*p* = 0.001; *p* = 0.019; *p* = 0.015, respectively). Adult-onset patients converted earlier than juvenile-onset OMG patients (*p* = 0.014). Upon multivariate logistic regression analysis, age of onset (Odds ratio [OR] 1.023, 95% confidence interval [CI] 1.006–1.041, *p* = 0.007) and positive facial nerve RNS (OR 2.826, 95%CI 1.045–5.460, *p* = 0.038) were found to be positively associated with generalized development. Moreover, an obviously negative association was found for disease duration (OR 0.603, 95%CI 0.365–0.850, *p* = 0.019).

**Conclusions:**

Age of onset, disease duration and facial nerve RNS test can predict generalized conversion of OMG under immunosuppressive therapy. Adult-onset, shorter disease duration and facial nerve RNS-positive OMG patients have a higher risk of generalized development.

## Background

Myasthenia gravis (MG) is an acquired neuromuscular junction disorder mainly mediated by pathogenic autoantibodies against acetylcholine receptors at the postsynaptic membrane. According to clinical manifestations, MG is classified as ocular MG (OMG) and generalized MG (GMG). Prior studies have shown that approximately 70% of MG patients initially present pure ocular symptoms and more than 90% of OMG patients undergo generalized conversion within 2–3 years [1, 2]. When systemic involvement of skeletal muscles is present, a diagnosis of GMG can be made and its outcome becomes worse [3–5]. Nowadays, it has been widely accepted that generalization of OMG is a classical phenomenon even though the conversion rate varies in different studies, and a series of predictors have been postulated to be correlated with secondary generalization. In Korean and Singapore populations, repetitive nerve stimulation (RNS) tests and anti-acetylcholine receptor antibody (AChR-Ab) have been identified as predictive factors for generalization of OMG [6, 7]. While in a Germany study, the presence of thymoma was deemed to be the only risk factor for secondary generalization [8]. Previous studies have suggested that immunosuppressive therapy reduce the conversion rate of OMG to 7–23.3% [6, 7, 9], but a proportion of patients still ineluctably experience secondary generalization, and the predictors of generalization in these patients require further investigation. This study attempts to explore the potential predictors of generalized conversion in OMG patients under immunosuppressive therapy.

## Methods

### Design, population and settings

A total of 223 OMG patients under immunosuppressive treatments in Tangdu Hospital from June 2008 to June 2012 were retrospectively reviewed. The OMG diagnosis was confirmed in a double-blinded manner by two attending physicians or higher according to initial symptoms of unilateral or bilateral ptosis, diplopia, or both and at least one of the followings: (1) unequivocal response to neostigmine, (2) positive RNS tests, and (3) seropositivity for AChR-Ab if available. The exclusion criteria included: (1) GMG symptoms concurrent with the onset of OMG or within the first month of disease [10], (2) neonatal myasthenia gravis [11], and (3) disagreed clinical diagnoses were presumed by two attending physicians or higher. All patients were treated with steroids alone or in association with other immunosuppressants including azathioprine, immunoglobulin and cyclosporine A (Table 1). Steroid treatments included intravenous glucocorticoid pulse therapy and oral prednisone at an initial dose of 0.75–1.0 mg/kg per day with slow tapering according to clinical condition. Intravenous immunoglobulin was administered at 0.4 g/kg per day for 5 consecutive days. Oral azathioprine was given at 50 mg–100 mg per day and cyclosporine A at 100 mg per day.

**Table 1:** Demographic features of 223 OMG patients

Variables	*n* (%)
Disease duration (y), *median* (IQR)	4 (2–6)
Gender	(*N* = 223)
Male	116 (52.0)
Female	107 (48.0)
Age of onset	(*N* = 223)
< 20 y	110 (49.3)
≥ 20 y	113 (50.7)
Initial symptoms	(*N* = 223)
Unilateral ptosis	110 (49.3)
Bilateral ptosis	40 (17.9)
Diplopia	10 (4.5)
Unilateral ptosis with diplopia	42 (18.8)
Bilateral ptosis with diplopia	21 (9.4)
Neostigmine test	(*N* = 171)
Positive	163 (95.3)
Negative	8(4.7)
Positive rate of RNS test	
Facial nerve	25/84 (29.8)
Axillary nerve	18/76 (23.7)
Ulnar nerve	2/74 (2.7)
Thyroid abnormality	(*N* = 102)
Hyperthyroidism	9 (8.8)
Subclinical hyperthyroidism	4 (3.9)
Hypothyroidism	2 (2.0)
Subclinical hypothyroidism	2 (2.0)
Thymus abnormality	(*N* = 174)
Thymoma	25 (14.4)
Thymic hyperplasia	7 (4.0)
Non-degraded thymus	7 (4.0)
Immunosuppressants	(*N* = 223)
Steroids	159 (71.3%)
Azathioprine	8 (3.6%)
Steroids + azathioprine	53 (23.8%)
Steroids + cyclosporine A	2 (0.9%)
Steroids + IVIg	1 (0.4%)

Abbreviations: *RNS* Repetitive nerve stimulation, *y* Year, *IQR* Interquartile range, *IVIg* Intravenous immunoglobulin. Unless otherwise noted, values are shown as *n* (%)

### Study protocol and data collection

OMG patients’ medical records were retrospectively reviewed and face-to-face interviews were conducted after written informed consent was obtained. Clinical variables including gender, age at onset, disease duration, clinical symptoms, response to neostigmine and RNS tests, thymus and thyroid examination were collected. When describing demographic features of the OMG patients, disease duration was defined as the interval from ocular symptom onset to the last follow-up irrespective of the presence of generalized conversion. In the last follow-up visit, a comprehensive interview was conducted to determine whether or not generalized conversion had occurred, which was defined as appearance of any systemic symptoms beyond extraocular muscle weakness such as dysphagia, dysarthria or weakness of extremities and even respiratory difficulties. Meanwhile, time to generalization was determined in the patients undergoing generalized conversion and re-identified as disease duration for those patients during the following logistic regression analysis for exploring the predictors of generalization.

### Statistical analysis

Data was presented as number with percentage or median with interquartile range (IQR) and statistical analysis was performed by SPSS19.0 software (SPSS Inc., Chicago, IL, USA). Differences of categorical variables between groups were evaluated by *χ*^2^ test and Fisher’s exact test when necessary. Manne-Whitney *U* test was used to analyze the difference in age of onset and generalized interval between groups. Probability of generalized conversion was presented using the Kaplan-Meier method and analyzed with log-rank test. Univariate logistic regression analysis was performed on variables of disease duration, age at onset, electrophysiological tests and thymic abnormalities. Multivariate logistic regression analysis was performed using variables with *p* < 0.100 during the univariate analysis to find the predictors of generalization of OMG. Odds ratio (OR) with 95% confidence intervals (CI) was calculated. A *p* value < 0.05 was considered statistically significant in all tests.

## Results

### Demographic features of OMG patients

A total of 223 OMG patients who completed the final follow-up were included in this retrospective study. The study population consisted of 116 males and 107 females, with a male-to-female ratio of 1.1:1. The median disease duration was 4 years (IQR, 2–6 years). The percentage of juvenile-onset patients (< 20 y) was 49.3% and of adult-onset patients (≥ 20 y) was 50.7% (Table 1). There were no significant differences in initial symptoms and complications including positive rates of thymoma between juvenile-onset and adult-onset patients (*p* > 0.05). The positive rate of neostigmine test of the two groups was 94.9% in juvenile-onset group and 95.7% in adult-onset group (*p* = 0.799). In juvenile-onset group, 23, 24 and 31 cases were examined with facial, axillary and ulnar RNS tests, respectively. And 61, 52 and 44 cases were examined with facial, axillary and ulnar RNS tests respectively in adult-onset group. While only the difference in the positive rate of axillary RNS test between the two groups showed statistical significance (*p* = 0.032) (Table 2).

**Table 2:** Comparison of clinical features between juvenile-onset and adult-onset OMG patients

Variables	< 20 y	≥ 20 y	*P* value
Gender (Male/Female)	110 (53/57)	113 (63/50)	0.258
Initial symptoms			
Unilateral ptosis	58/110 (52.7%)	52/113 (46.0%)	0.316
Bilateral ptosis	20/110 (18.2%)	20/113 (17.7%)	0.925
Diplopia	2/110 (1.8%)	8/113 (7.1%)	0.102
Unilateral ptosis with diplopia	18/110 (16.4%)	24/113 (21.2%)	0.352
Bilateral ptosis with diplopia	12/110 (10.9%)	9/113 (8.0%)	0.452
Neostigmine test (+)	74/78 (94.9%)	89/93 (95.7%)	0.799
Positive rate of RNS test			
Facial nerve	10/23 (43.5%)	15/61 (24.6%)	0.091
Axillary nerve	2/24 (8.3%)	16/52 (30.8%)	0.032
Ulnar nerve	0/31	2/44 (4.5%)	–
Thyroid abnormality	5/42 (11.9%)	12/60 (20.0%)	0.280
Hyperthyroidism	2/5 (40%)	7/12 (58.3%)	0.620
Subclinical hyperthyroidism	2/5 (40%)	2/12 (16.7%)	0.538
Hypothyroidism	1/5 (20%)	1/12 (8.3%)	0.515
Subclinical hypothyroidism	0/5	2/12 (16.7%)	–
Thymus abnormality	12/76 (15.8%)	27/98 (27.6%)	0.065
Thymoma	7/12 (58.3%)	18/27 (66.7%)	0.723
Thymic hyperplasia	3/12 (25.0%)	4/27 (14.8%)	0.654
Non-degraded thymus	2/12 (16.7%)	5/27 (18.5%)	1.000

Abbreviations: *RNS* Repetitive nerve stimulation, *y* Year. Statistical analysis was performed by *χ2* test and Fisher’s exact test when necessary

### Conversion of OMG to GMG

Thirty-eight out of 223 OMG patients (17.0%; 18 males and 20 females) progressed to GMG with a median interval of 0.9 years (IQR, 0.3–2.1 years). The median age of onset was 46 years (IQR, 37.5–55 years) in male patients and 30.5 years (IQR, 8–49.8 years) for female patients, and no significant difference was seen between the two groups (*p* = 0.118). Among the 38 patients undergoing generalization, 21 (55.3%; 8 males and 13 females) progressed to type IIA according to the Osserman classification. Nine male and 6 female patients conversed to type IIB. The remaining 1 male and 1 female patients progressed to type III. No significant difference was observed in the distribution of conversion types between the two groups (*p* = 0.512) (Table 3).

**Table 3:** Gender comparison of clinical features in 38 patients with generalized conversion

Variables	Male	Female	*P* value
Patients with conversion, *n* (%)	18 (47.4)	20 (52.6)	–
Age at onset (y), *median* (IQR)	46 (37.5–55)	30.5(8–49.8)	0.118
Time to generalization (y), *median* (IQR)	0.8 (0.2–2.0)	0.9 (0.3–2.9)	0.692
Osserman classification, *n*			
IIA	8	13	
IIB	9	6	
III	1	1	0.512

Abbreviations: *y* Year, *IQR* Interquartile range. Statistical analysis was performed by Manne-Whitney *U* test for age at onset and time to generalization, and by Fisher’s exact test for Osserman classification between subgroups

### Probability of conversion to GMG

**Fig. 1:**
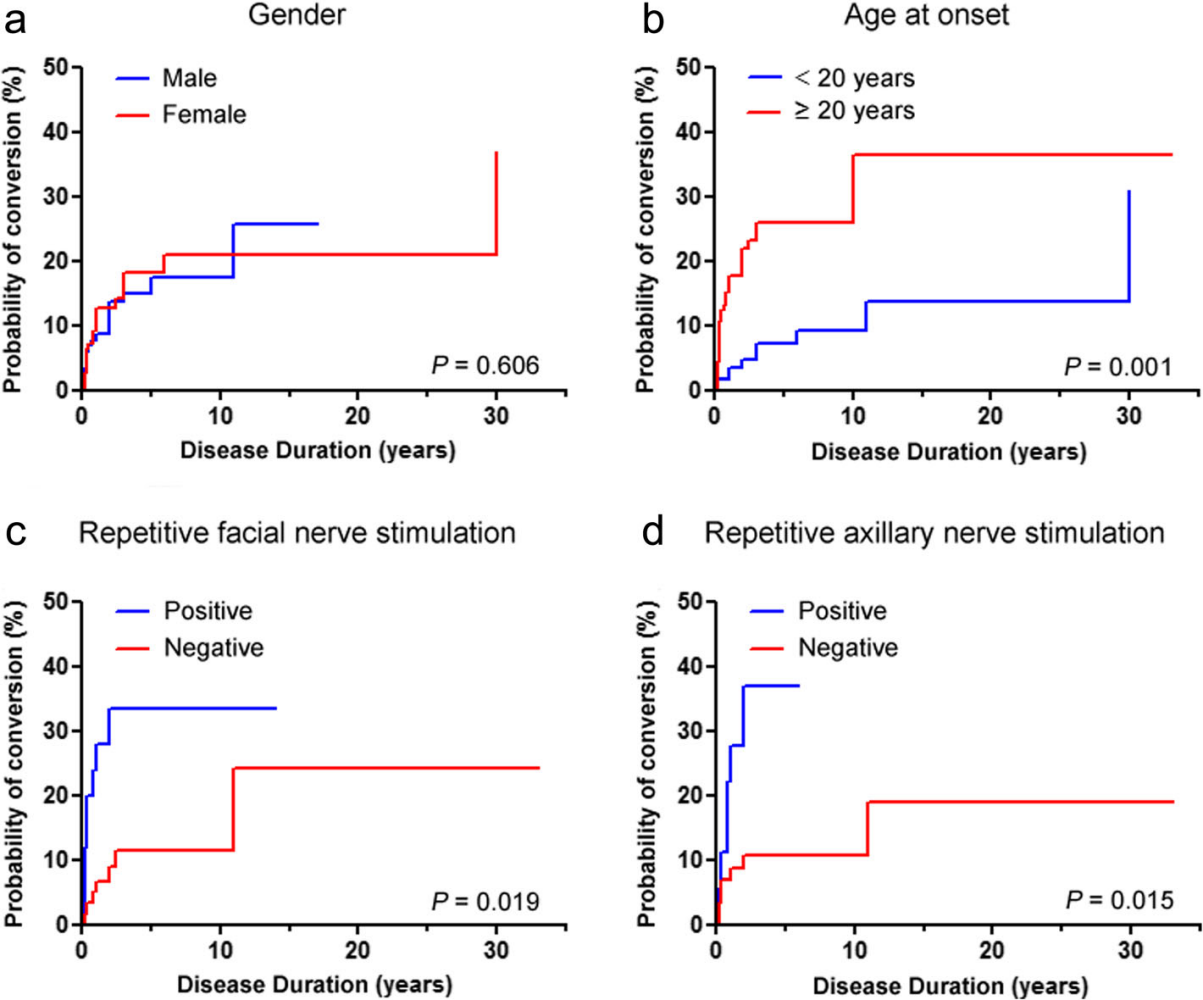
**a** Kaplan-Meier curve depicting probability of conversion over time (years) from ocular to generalized myasthenia gravis between male and female patients. **b** Kaplan-Meier curve depicting probability of conversion over time (years) from ocular to generalized myasthenia gravis between juvenile-onset and adult patients. **c** Kaplan-Meier curve depicting probability of conversion over time (years) from ocular to generalized myasthenia gravis in patients with positive facial nerve RNS compared to those negative. **d** Kaplan-Meier curve depicting probability of conversion over time (years) from ocular to generalized myasthenia gravis in patients with positive axillary nerve RNS compared to those negative. *p* < 0.05 indicates statistically significance

**Fig. 2:**
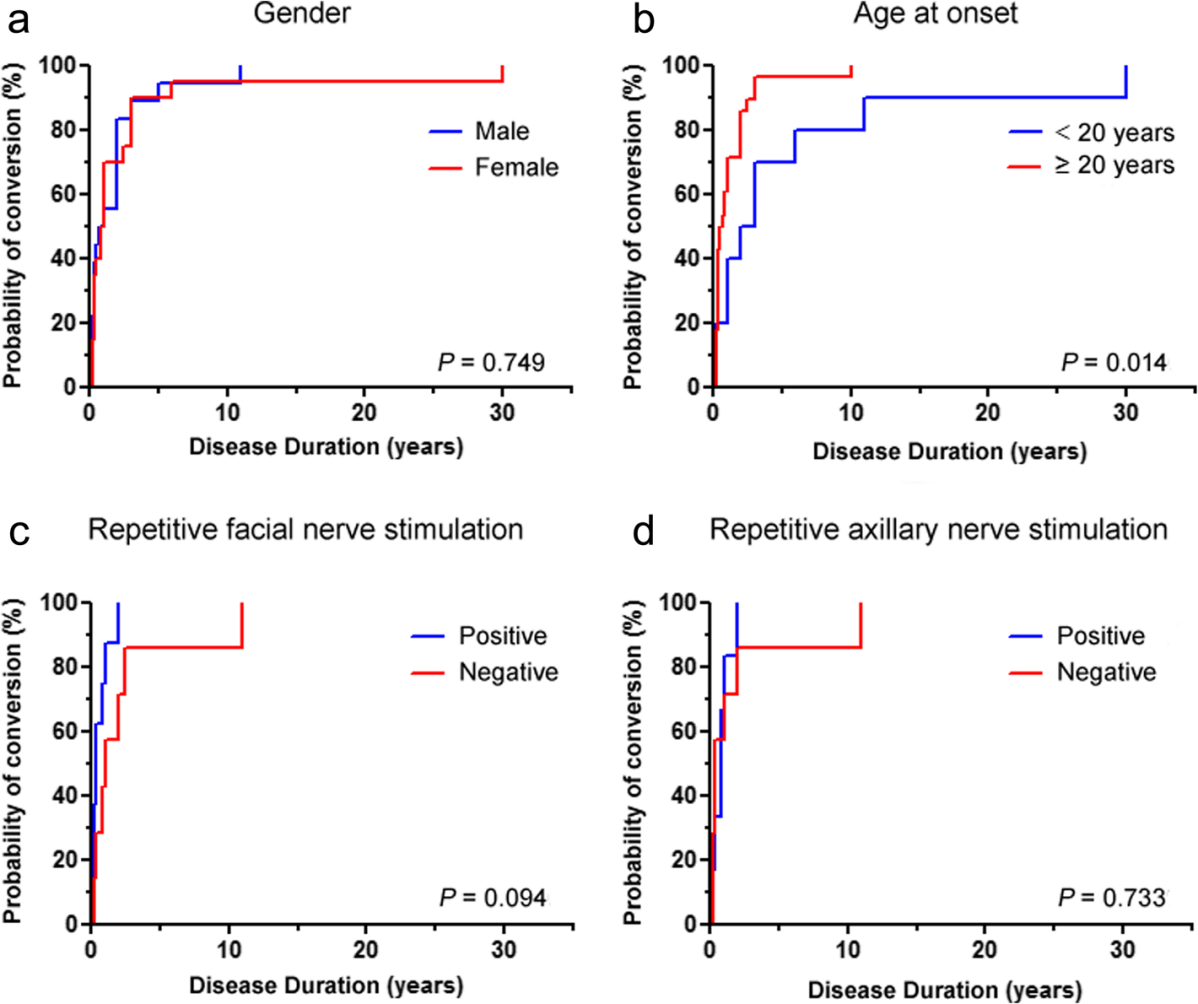
**a** Kaplan-Meier curve depicting conversion tempo between male and female patients of 38 generalized. **b** Kaplan-Meier curve depicting conversion tempo between juvenile-onset and adult patients of 38 generalized. **c** Kaplan-Meier curve depicting conversion tempo in 38 generalized patients who had positive facial nerve RNS compared to those negative. **d** Kaplan-Meier curve depicting conversion tempo in 38 generalized patients who had positive axillary nerve RNS compared to those negative. *p* < 0.05 indicates statistically significance

Kaplan-Meier method was used to obtain cumulative probabilities for conversion from OMG to GMG. No significant difference was observed between males and females (*p* = 0.606), but a significantly higher probability of conversion to GMG was found in adult-onset OMG patients than juvenile-onset OMG patients (*p* = 0.001). Similarly, patients with positive facial or axillary nerve RNS tests had a significantly higher probability of conversion to GMG than those with negative RNS tests (*p* = 0.019 and 0.015, respectively) (Fig. 1). We further assessed the intervals from OMG onset to conversion in the 38 patients undergoing generalization. Adult-onset patients had an obviously shorter time to generalization than juvenile-onset patients (*p* = 0.014), but no significant differences were observed between male and female patients (*p* = 0.749), patients with positive and negative facial nerve RNS tests (*p* = 0.094) and patients with positive and negative axillary nerve RNS tests (*p* = 0.733), respectively (Fig. 2).

### Predictors of conversion to GMG

Clinical variables including gender, age at onset, disease duration, positive facial or axillary nerve RNS and thymic abnormalities were selected for univariate logistic regression analysis to explore the potential predictors of generalization. Among these, age at onset (Odds ratio [OR] 1.025; 95% confidence interval [CI] 1.009–1.041; *p* = 0.002), disease duration (OR 0.783; 95%CI 0.651–0.906; *p* = 0.004), positive facial nerve RNS (OR 3.496; 95%CI 1.103–11.409; *p* = 0.033) and positive axillary nerve RNS (OR 3.643; 95%CI 1.012–13.049; *p* = 0.044) significantly predicted GMG conversion except gender and thymic abnormalities. Further multivariate logistic regression analysis revealed that age at onset (OR 1.023, 95%CI 1.006–1.041, *p* = 0.007) and positive facial nerve RNS (OR 2.826, 95%CI 1.045–5.460, *p* = 0.038) were positively associated with generalized conversion. Moreover, an obviously negative association was found for disease duration (OR 0.603, 95%CI 0.365–0.850, *p* = 0.019) (Table 4).

**Table 4:** Logistic regression analysis of predictors of conversion to GMG

	OR	95%CI	*P* value
Univariate logistic regression *Variables*			
Gender	1.252	0.621–2.537	0.529
Age at onset	1.025	1.009–1.041	0.002
Disease duration	0.783	0.651–0.906	0.004
Positive facial nerve RNS	3.496	1.103–11.409	0.033
Positive axillary nerve RNS	3.643	1.012–13.049	0.044
Thymic abnormalities	2.259	0.949–5.202	0.058
Multivariate logistic regression *Variables*			
Age at onset	1.023	1.006–1.041	0.007
Disease duration	0.603	0.365–0.850	0.019
Positive facial nerve RNS	2.826	1.045–5.460	0.038

Abbreviations: *RNS* Repetitive nerve stimulation, *OR* Odds ratio, *CI* Confidential interval

## Discussion

MG is a heterogeneous disease affected by ethnicity, gender, age at onset, disease duration and other factors. Generalized conversion is a typical feature for MG patients who initially present pure ocular symptoms, and approximately 90% of OMG patients from Caucasian populations might progress to GMG within the first 2–3 years [1, 2]. However in this study enrolling OMG patients from Northwest China, only 38 of 223 patients (17.0%) converted to GMG over a median disease course of 4 years, which was similar to previously reported figures (11.4%-29%) in Asian populations from Southern China, Hong Kong, Korean and Singapore [6, 7, 12–14]. This discrepancy might be related to differential MG susceptibility at different ages. It is generally accepted that adults from Caucasian populations are more vulnerable to MG [15], but in Asian populations juvenile MG accounts for more than half of the MG patients [13]. Given that older age at onset has been demonstrated to correlate with higher risk of generalization [16, 17], the lower conversion rate in this study than in Caucasian population further highlights the potential role of ethnicity in the prevalence of OMG.

Several studies suggested that factors such as age at onset, AChR-Ab titers, positive rate of RNS, thymoma and immunosuppressive therapy were associated with generalization [6–9]. Immunosuppressive therapy has been given to OMG patients with intention to reduce the risk of secondary generalization [2, 4, 18], but it is noted that a small proportion of patients still underwent generalized conversion [19] and the possible predictors remain unknown. In this study, we enrolled the OMG patients who received steroids and/or other immunosuppressants and found that 17.0% converted to GMG eventually. The conversion rate was consistent with previous studies where immunosuppressed and non-immunosuppressed groups were compared [6, 7, 19, 20], and also suggested that the data was qualified for investigation of generalization of OMG under immunosuppressive therapy.

Age at onset has been identified as one of the risk factors for generalization of OMG. Prior studies showed that the conversion rate of juvenile-onset OMG fluctuated between 23 and 43% [3, 21], while that of adult-onset OMG between 31 and 49% [4, 22]. Kamarajah and Wang reported that late-onset was correlated with higher risk of generalization [16, 17]. In another study, patients were inclined to convert to GMG with increasing age at onset [2]. In this study, OMG patients under immunosuppressive treatments were divided into juvenile-onset OMG and adult-onset OMG subgroups. No significant differences were observed between the subgroups in clinical variables of interest except for axillary nerve RNS test. In Kaplan-Meier curve analysis, adult-onset OMG patients had a higher cumulative probability of conversion to GMG than juvenile-onset patients. Moreover, adult-onset patients had a shorter time to generalization than juvenile-onset patients. Further univariate and multivariate logistic regression analyses revealed that age at onset was positively associated with secondary generalization. Similar to previous studies [2, 16, 17], our findings confirmed the predictive value of age at onset in generalization of OMG under immunosuppressive therapy.

Positive RNS test is observed in a portion of OMG patients [23] and it is controversial whether positive RNS results predict secondary generalization. Some studies showed that positive RNS is associated with higher conversion rate [6, 7], whereas others did not support this [1, 4, 6, 24]. In this study, positive facial RNS test was proved to be one predictor of secondary generalization. Although the association between axillary RNS test and risk of generalization was found in both Kaplan-Meier curve and univariate logistic regression analyses, but no clear association was evidenced by the multivariate logistic regression analysis, probably due to the limited number of patients who received axillary nerve RNS test. The predictive value of axillary nerve RNS test needs to be investigated in further studies with larger sample size and longer follow-up period.

Some scholars pointed out that positive AChR-Ab and abnormal single-fiber electromyography (SFEMG) tests in the early stages of the disease could predict more generalized conversion [9, 25, 26], but the predictive values of these variables were not confirmed by other studies [8, 27]. Unfortunately, we were unable to detect AChR-Ab titers and perform SFEMG due to restrictions of examination methods, but this variable was strongly recommend to be included in future studies. Thymic abnormalities, especially thymoma has been considered one predictor of secondary generalization [8]. However in our cohort, thymic abnormality was excluded as a potential predictor during logistic regression analysis. This might be interpreted to some extent by early application of immunosuppressive therapy since it could weaken or eliminate the effect of thymic abnormalities in promoting generalized conversion. In addition, studies have shown that the longer disease duration the lower conversion risk of OMG [19, 26, 28]. By multivariate logistic regression analysis, we found that disease duration was negatively associated with generalization in the OMG patients receiving immunosuppressive therapy. The similar findings suggested that immunosuppressive treatments could not hamper the effect of disease duration on secondary generalization.

There are some limitations in this retrospective study. Restricted clinical condition and examination methods, unwillingness of partial patients and missing data from medical records resulted in small sample size of clinical variables, especially RNS tests in our cohort, but the percentage of patients who entered the final statistical analysis and of those with positive RNS tests were comparable to other studies [7, 24, 29]. This supported that the conclusions from statistical analysis in this study were reliable. In the future, prospective studies with rigorous design and complete data as well as with larger sample size and longer follow-up period are recommended to obtain more convincing results and conclusions.

## Conclusions

Immunosuppressive therapy can reduce, but not avoid generalization of OMG. Among the patients who receive immunosuppressive treatments, age at onset, disease duration and positive facial nerve RNS are identified as the predictive factors of generalized conversion. Adult-onset, shorter disease duration and positive facial nerve RNS indicate the higher risk of secondary generalization. Further prospective studies with larger sample size and longer follow-up period are needed to corroborate the conclusions pertaining to this study.

## Data Availability

The data sets in this study are available from the corresponding author on reasonable request.

## Abbreviations

MG: Myasthenia gravis
OMG: Ocular myasthenia gravis
GMG: Generalized myasthenia gravis
RNS: Repetitive nerve stimulation
OR: Odds ratio
CI: Confidence interval
AChR-Ab: Anti-acetylcholine receptor antibody
IQR: Interquartile range
y: Year
